# Eukaryotic microorganisms in cold environments: examples from Pyrenean glaciers

**DOI:** 10.3389/fmicb.2013.00055

**Published:** 2013-03-19

**Authors:** Laura García-Descalzo, Eva García-López, Marina Postigo, Fernando Baquero, Alberto Alcazar, Cristina Cid

**Affiliations:** ^1^Microbial Evolution Laboratory, Centro de Astrobiología (Consejo Superior de Investigaciones Cientificas - Instituto Nacional de Técnica Aeroespacial)Torrejón de Ardoz, Madrid, Spain; ^2^Department of Microbiology, Hospital Ramon y CajalMadrid, Spain; ^3^Department of Investigation, Hospital Ramon y CajalMadrid, Spain

**Keywords:** eukaryotic microorganisms, glaciers, ancient ice, climate change, biodiversity, Pyrenean glaciers

## Abstract

Little is known about the viability of eukaryotic microorganisms preserved in icy regions. Here we report on the diversity of microbial eukaryotes in ice samples derived from four Pyrenean glaciers. The species composition of eukaryotic communities in these glaciers is unknown mostly because of the presence of a multi-year ice cap, and it is not clear whether they harbor the same populations. The recent deglaciation of these areas is allowing an easy access to glacial layers that correspond to the “Little Ice Age” although some isolated deposits are attributed to previous glacial cycles. In this study, we use molecular 18S rRNA-based approaches to characterize some of the microbial eukaryotic populations associated with Pyrenean glaciers. Firstly, we performed a chemical and microscopical characterization of ice samples. Secondly, molecular analyses revealed interesting protist genetic diversity in glaciers. In order to understand the microbial composition of the ice samples the eukaryotic communities resident in the glacial samples were examined by amplifying community DNA and constructing clone libraries with 18S rRNA primers. After removal of potential chimeric sequences and dereplication of identical sequences, phylogenetic analysis demonstrated that several different protists could be identified. Protist diversity was more phylum rich in Aneto and Monte Perdido glaciers. The dominant taxonomic groups across all samples (>1% of all sequences) were Viridiplantae and Rhizaria. Significant variations in relative abundances of protist phyla between higher and lower glaciers were observed. At the genus level, significant differences were also recorded for the dominant genera *Chloromonas*, *Raphidonema*, *Heteromita*, *Koliella*, and *Bodomorpha*. In addition, protist community structure showed significant differences between glaciers. The relative abundances of protist groups at different taxonomic levels correlated with the altitude and area of glaciers and with pH of ice, but little or no relationships to other chemical characteristics were found.

## INTRODUCTION

Small eukaryotes are probably the most abundant eukaryotes on Earth. They have been found in all extreme environments (Caron et al., 1999), addressing a wide range of temperatures from tropical oceans (Li et al., 1994) to polar sea ice (Bachy et al., 2011).

The discovery of cold-tolerant microorganisms in glaciated and permanently frozen environments has broadened the known range of environmental conditions which support microbial life. Although these microorganisms that inhabit permanently cold ecosystems (representing one of the largest biosphere reserves on Earth) have been studied only for their ability to survive in such extreme conditions, recent studies have provided evidence that these habitats (deep sea, Polar Regions, mountain glaciers, etc.) can be colonized by both obligate and facultative psychrophilic microorganisms (Alcazar et al., 2010). Such ecosystems represent one of the last unexplored frontiers of ecology, and psychrophilic microbial populations sharing such habitats constitute an important part of cold-adapted biodiversity and play an essential role as nutrient cyclers and organic matter mineralizers.

It is difficult to characterize these organisms by simple observation with optical microscopy, and cultivation methods do not allow all the organisms to grow. Pigment and/or fatty acid analysis can provide some information on the structure and dynamics of the phototrophic and/or heterotrophic behavior of small eukaryotes, but the phylogenetic information supplied by these methods is limited (Lefranc et al., 2005). During the last decade, molecular techniques have greatly increased our knowledge by identifying the smallest organisms. Environmental studies of eukaryotic diversity based on polymerase chain reaction (PCR) amplification, cloning, and sequencing of the 18S rRNA gene have revealed a wide diversity of protists in a variety of poorly explored habitats (Bachy et al., 2011). Despite the power of molecular ecology techniques, these methods have not been as widely used for microeukaryotes as for prokaryotes. Several recent studies have analyzed the diversity of small eukaryotes, sampled in different cold ecosystems, by gene cloning and sequencing of rRNA genes and have shown high phylogenetic diversity (Lefranc et al., 2005). These studies identified a wide variety of lineages and retrieved sequences not clearly assigned to any known organisms. It should be emphasized that most of these studies on small eukaryote diversity were conducted in marine and lake ecosystems (Medinger et al., 2010; Bachy et al., 2011). Thus, little is known about the diversity of microorganisms from glaciers.

There have been a number of studies on the microbiological composition of such psychrophilic populations in recent years. Viable bacterial communities have been observed beneath glaciers in the northern (Skidmore et al., 2000) and southern hemisphere (Foght et al., 2004). With reference to eukaryotic microorganisms, de García et al. (2007) described the occurrence of viable yeasts in meltwaters running off glaciers of northwest Patagonia, and Butinar et al. (2007) isolated culturable yeasts from basal ice layers of high arctic glaciers of the Svalbard Islands. Likewise, Margesin et al. (2007) and Turchetti et al. (2008) described the existence of yeasts in some Alpine glacier habitats (e.g., cryoconites, ice cores, sediments, and meltwaters) and Branda et al. (2010) in Calderone glacier, Apennines. However, despite the profusion of results, studies on microbial eukaryotic communities harboring in cold habitats of Pyrenean area are so far lacking.

The global warming effect on microorganisms from glaciers is relevant since these organisms constitute the base of the food webs and a significant number of protists beneath glaciers are reported to play important roles in chemical weathering and carbon cycling processes (Dolhi et al., 2012). It is well known that worldwide glaciers are strongly retreating due to ongoing climate change. In this context, the Mediterranean region represents a particularly delicate area, where glaciers of limited size are placed in mountain chains of relatively low altitude (the Pyrenees, Atlas Mountains, Maritime Alps, Apennines). The Pyrenees hosts the only active glaciers that persist today in the Iberian Peninsula. In the early twentieth century, they occupied an approximate area of 3,300 ha, but currently, only cover about 390 ha (Arenillas et al., 2008). In this extension, approximately 58% (some 206 ha) is located in the Spanish side. These glaciers are a present source of concern because of their steady decline linked to global warming. With the disappearance of the Corral del Veleta glacier (Sierra Nevada, Spain) in 1913 (Gómez et al., 2003), these glaciers, along with a small residual nucleus (Calderone glacier) that is conserved in the Apennines became the southernmost in Europe. If the present trend continues, these glaciers might soon share the fate of Corral del Veleta. Accordingly, the study of psychrophilic microbial populations sharing such vanishing cold habitat is of increasing scientific interest.

Assessments of diversity and distribution of microorganisms in glaciers are fundamental to studies related to global warming. Our knowledge about the microorganisms living in mountain glaciers is still rudimentary mostly because of logistical challenges imposed by its inhospitable climate and the presence of a multi-year ice cap. Glacial ice is unique matrix that is capable of protecting microorganisms from long-term preservation. The organisms entrapped in glacial ice can provide information on evolutionary processes and ancient biodiversity (Ma et al., 2000). Global warming and associated environmental changes are predicted to have strong impacts on high-altitude ecosystems (IPCC, 2007), and the European mountains have already experienced an increase in temperature of 1–2°C during the twentieth century (Beniston et al., 1997; Djukic et al., 2012). A possible consequence of permafrost thawing, based upon predicted global warming scenarios, is that there may be an increase in microbial activity and an increase in active layer thickness (Gilichinsky and Wagene, 2006). Current atmospheric warming will have severe consequences for the structure and functioning of glacier ecosystems with changes that, in turn, may feed back on the global-scale composition of the atmosphere (Michelsen et al., 2012; Wagner et al., 2012). Here we report the diversity and distribution of microbial eukaryotes in four Pyrenean glaciers studied by 18S rRNA gene libraries and addresses some interesting questions: (i) what is the effect of altitude and glacier area on the composition of the microbial community? (ii) taking into account that ice melting is more dramatic in lower glaciers, which are the environmental differences associated to ice melting that affect microbial community? (iii) is it possible to discriminate between the effect on microbial community of area/altitude and the effect of chemical parameters? (iv) is it possible to find a microbial community or species to be used as an indicator of glacier retreat?

## MATERIALS AND METHODS

### SAMPLE COLLECTION AND PROCESSING

**FIGURE 1:**
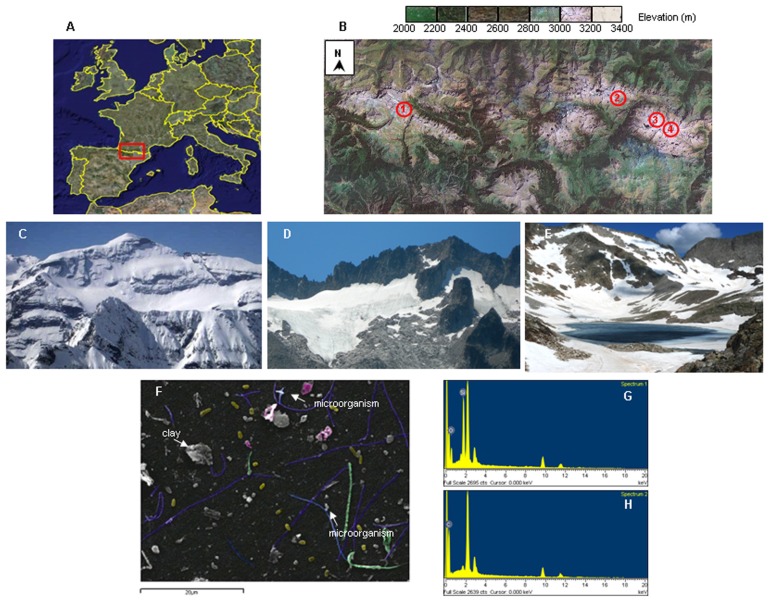
**Geologic setting of ice from Pyrenean glaciers and evidence of encased microorganisms.** (A,B) Map showing the distribution of ice samples collected at various locations in Monte Perdido (1), Literola (2), Maladeta (3), and Aneto (4) glaciers. **(C–E)** Photographs of Monte Perdido **(C)**, Aneto–Maladeta **(D)**, and Literola **(E)**. **(F)** Scanning electron microscopy image of microorganisms in a matrix of fine sediment particles from LIT ice meltwater illustrating their morphology compared with glacial till. **(G,H)** Representative energy-dispersive X-ray analysis spectra from microorganisms collected at Pyrenean glaciers. Full vertical scales in counts per second are variable according to the area analyzed. Horizontal spectral ranges are in keV.

Glacial ice samples were collected at four sites on the northern slope of Pyrenees: Aneto glacier (ANE), Maladeta glacier (MAL), Monte Perdido glacier (PER), and Literola glacier (LIT) in August, 2010 (**Figure 1**). These glaciers are located at altitudes of 3,404, 3,035, 3,355, and 2,740 m, and their surface are 64, 31, 32, and 1.5 ha, respectively (Rene, 2007; Arenillas et al., 2008). Ice samples were obtained by removing 20–30 cm of thick debris and cutting out a square block of 20 cm on a side. Three sampling replicates were collected from each glacier. Samples were wrapped in plastic bags and stored at −20°C until processing. Ice samples were processed by using a surface decontamination and melting procedure consistent with previous studies (Bidle et al., 2007). A section of block ice was removed from −20°C and soaked in ice-cold 95% ethanol for 1 min, followed by extensive rinsing with 0.22 μm-filtered MilliQ water, effectively ablating the exterior 3-cm shell of ice samples (corresponding to 30% of total ice volume). These procedures were effective at removing surface contamination from inner shell ice samples (Rogers et al., 2004; Christner et al., 2005). The decontaminated interior ice was thawed in a sterile plastic bag at 4°C and used for analyses. To control for laboratory contamination, 1 l of MilliQ rinse water was frozen, thawed, filtered onto polycarbonate filters, and subjected to identical analytical procedures. All procedures were performed by using bleach-sterilized work areas, a UV-irradiated laminar flow hood, ethanol-sterilized tools, and sterilized gloves.

### CHEMICAL ANALYSIS OF MELTWATER

Basic measurements of physical and chemical parameters of meltwater from various sites were made with a temperature-calibrated pH, conductivity, and salinity meter (WTW, Weilheim, Germany). Assays for dissolved inorganic nitrogen (${NH}_{4}^{+}$, $NO_{2}^{-}$, and $NO_{3}^{-}$) were performed by ion chromatographic method using suppressed conductivity detection in a 861 Advance Compact IC system (Metrohm AG, Herisau, Switzerland). Chromatograms were recorded using the Metrohm IC Net 2.3 SR4 software. The system was run in the isocratic mode with the column at 45°C.

### SCANNING ELECTRON MICROSCOPY

Samples (50 ml volume) for scanning electron microscopy (SEM) observation were filtered in the lab (0.22 μm Millipore filters), preserved in 2% glutaraldehyde, rinsed in 0.22 μm-filtered 50 mM phosphate buffer, and dehydrated using an ascending (30, 50, 70, 90, and 100%) series of ethanol. Filter disks were then air dried overnight and mounted on SEM stubs with carbon pads and sputter coated with gold-palladium for 1.5 min at 15 mA voltage. Observation and imaging were examined using a Jeol 5600LV scanning electron microscope with an INCA Oxford auxiliary X-ray energy-dispersive spectroscopy microanalytical system.

### DNA EXTRACTION AND PCR AMPLIFICATION

Approximately 400 ml of each frozen sample was melted at 4°C and filtered through a 0.22-μm filter (Millipore). Community DNA was extracted using the GNOME BIO101 kit (MP Biomedicals, Illkirch, France) and purified with QIAquick PCR purification kit (Qiagen, Hilden, Germany) according to manufactures’ instructions. Extraction procedures were identical for all ice samples. The 18S rRNA genes from mixed microbial DNA were amplified by PCR. Near full-length 18S rDNA fragments were amplified by PCR using the eukaryotic-specific primers 1F (CTG GTT GAT CCT TGC CAG; Lefranc et al., 2005) and 502R (ACC AGA CTT GCC CTC C; Amann et al., 1990). PCR was carried out under the following conditions: 33 cycles (denaturation at 94°C for 30 s, annealing at 46°C for 30 s, extension at 72°C for 35 s), was preceded by 5 min denaturation at 94°C, and followed by 7 min extension at 72°C. PCR was optimized by both diluting the template and by increasing the number of thermal cycles.

To control for false-positive PCR signals, 1 l of MilliQ water was frozen, thawed, and subjected to the same DNA extraction procedure. This material was used as a template with the specific primers to test for contamination and PCR artifacts.

### SEQUENCING AND PHYLOGENETIC ANALYSIS

18S rDNA PCR amplicons were cloned using TOPO TA Cloning Kit (Invitrogen, Carlsbad, CA, USA), and plasmid DNA (112 ANE clones, 35 MAL clones, 108 PER clones, and 44 LIT clones) was bidirectionally sequenced with a 3730XL sequencer (Applied Biosystems). Chimeric sequences were identified with the CHIMERA-CHECK program (Ribosomal Database Project II, Michigan State University, East Lansing, MI, USA) and discarded.

Sequences were analyzed using BLAST at the NCBI database^1^. Representative sequences were aligned using the software of ClustalX (version 2.0; Larkin et al., 2007), the results were corrected manually and alignment uncertainties were omitted in the phylogenetic analysis. Their phylogenetic relationship was analyzed using the software MEGA 5 (Tamura et al., 2011) and the ARB software (Ludwig et al., 2004) with parsimony, neighbor-joining, and maximum likelihood analyses. In all cases, general tree topology and clusters were stable, xxand reliability of the tree topologies was confirmed by bootstrap analysis using 1,000 replicate alignments. A consensus tree was generated.

Rarefaction analysis was performed using Analytic Rarefaction software (version 1.3)^2^, based on previous analytic solutions (Raup, 1975; Tipper, 1979).

### STATISTICAL ANALYSIS

Statistical differences on the number of clones, number of operational taxonomic units (OTUs) and Shannon–Wiener index were studied by analysis of variance (ANOVA) test. Data of OTUs and clones are media values of three sampling replicates. Relationships between the number of taxa found in glaciers and environmental variables were analyzed by linear regression analysis (Pearson’s correlation coefficient *r*).

Abundance-based coverage estimator (ACE) and Chao1 (Chao and Bunge, 2002) indices were calculated using the EstimateS program (version 8.2.0)^3^ (Colwell, 2011).

Effects of environmental variables on the community composition were investigated by a combination of analysis developed with CANOCO version 4.5 software (Microcomputer Power, Ithaca; Jongman et al., 1995). For statistical analysis, Monte Carlo permutation tests with 500 permutations were used.

### NUCLEOTIDE SEQUENCE ACCESSION NUMBERS

Sequences obtained in this study have been deposited in the EMBL sequence database under accession numbers JX196712 and JX456225 to JX456234.

## RESULTS

### GENERAL CHARACTERISTICS OF THE ICE SAMPLES AND CHEMICAL PROPERTIES

In this study, we assessed and compared the composition of eukaryotic microorganisms present in samples from four Pyrenean glaciers. ANE, MAL, PER, and LIT contained a broad size spectrum of particles and sand debris as the ice contained a layer of sand and organic matter of aeolian origin below the surface. These inorganic particles contributed to variations in chemical properties between the meltwater samples. Meltwater of ANE and MAL were pH 6.7 and 6, respectively, whereas those from PER and LIT were 4.8 and 5.7, possibly due to the chemical reactions of fine particles of, e.g., pyrite (**Table 1**). These fine particles were analyzed in a spectroscopy microanalytical system in order to exactly define whether the observed particles corresponded to microorganisms or inorganic material (**Figures 1G,H**). Spectrum 1 (**Figure 1G**) contains two major peaks corresponding to silicon and oxygen, demonstrating it is a clay mineral, moreover spectrum 2 (**Figure 1H**) shows a major peak of carbon.

**Table 1:** Chemical analysis of ice meltwater from ANE, MAL, PER, and LIT.

Sample	pH	Salinity (ppt)	${NH}_{4}^{+}$ (μM)	$NO_{2}^{-}$ (μM)	$NO_{3}^{-}$ (μM)
ANE-1	6.7	0.23	1.8	4.0	5.4
ANE-2	6.7	0.27	2.3	3.1	3.9
ANE-3	6.8	0.21	2.2	3.2	4.3
Average (SD)	6.7 (0.06)	0.24 (0.03)	2.1 (0.26)	3.4 (0.49)	4.5 (0.77)
MAL-1	6.5	0.20	4.0	4.8	6.5
MAL-2	6.2	0.29	3.1	5.3	7.2
MAL-3	5.3	0.23	4.3	4.9	7.0
Average (SD)	6.0 (0.51)	0.24 (0.04)	3.8 (0.51)	5.0 (0.22)	6.9 (0.29)
PER-1	4.9	0.15	7.5	3.5	6.1
PER-2	5.0	0.21	5.9	4.1	3.9
PER-3	4.6	0.19	6.4	3.8	4.2
Average (SD)	4.8 (0.21)	0.18 (0.03)	6.6 (0.82)	3.8 (0.30)	4.7 (1.19)
LIT-1	5.5	0.35	5.5	68.1	110.5
LIT-2	5.9	0.28	6.8	76.3	101.6
LIT-3	5.8	0.31	4.9	75.2	98.5
Average (SD)	5.7 (0.21)	0.31 (0.06)	5.7 (0.97)	73.2 (4.45)	103.5 (6.23)

In addition, ice properties such as salinity, pH, ${NH}_{4}^{+}$, $NO_{2}^{-}$, and $NO_{3}^{-}$ contents were determined (**Table 1**). The ice samples had overall low salinity and highly variable $NO_{2}^{-}$ and $NO_{3}^{-}$ contents ranging from 3.4 μM in ANE to 73.2 μM in LIT for $NO_{2}^{-}$ and ranging from 4.5 μM in ANE to 103.5 μM in LIT for $NO_{3}^{-}$ . Further, ${NH}_{4}^{+}$ presented a moderate variability between 2.1 μM in ANE and 6.6 μM in PER (**Table 1**). Generally, samples from higher glaciers showed lower amounts of ion concentrations, as salt solubility decreases in cold environments.

### 18S rRNA GENE CLONE LIBRARIES

**FIGURE 2:**
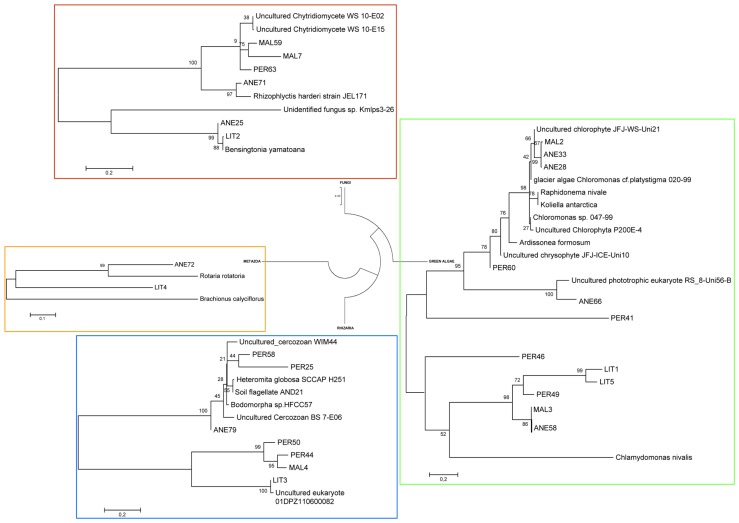
**Phylogenetic analysis of microbial community DNA in ice samples**. Consensus phylogenetic tree derived from 18S rRNA gene sequence data showing the four groups of microbial eukaryotes found in Pyrenean glaciers. The distance corresponding to one base change per hundred nucleotide positions is indicated by the scale bar. Accession numbers for the sequences used to make this tree are given in **Table 2**. Names in capital letters (ANE, MAL, PER, and LIT) correspond to clones retrieved in this study.

In order to understand the microbial eukaryotic populations of the ice samples, we amplified community DNA and constructed clone libraries with eukaryotic-specific 18S rRNA primers (**Table 2**). After removal of potential chimeric sequences and dereplication of identical sequences, phylogenetic analysis demonstrated that 31 different phylotypes could be identified (10 from 112 ANE clones, 7 from 35 MAL clones, 9 from 108 PER clones, and 5 from 44 LIT clones; **Figure 2**). Most of BLAST analysis of the 31 phylotypes (**Table 2**) revealed no identical clones to 18S rDNA sequences in GenBank, to currently cataloged species. Overall, the ANE, MAL, and PER clone libraries showed a high diversity (**Table 3**; **Figure 2**) with sequences belonging to Viridiplantae, Stramenopiles, Fungi, Rhizaria, and Metazoa divisions.

**Table 2:** Analysis of eukaryotic 18S rRNA clones retrieved from ANE, MAL, PER, and LIT ice samples.

Number	Clone or group name	Mean no. of clones in glacier	Accession number	Closest sequence match from BLAST search	Similarity (%)	Taxon
1	ANE25	2	AF289921	Unidentified fungus sp. Kmlps3-26	98	Fungi; environmental samples
2	ANE28	28	AF514406	*Chloromonas* sp. 047-99	99	Viridiplantae; Chlorophyta; Chlorophyceae; Chlamydomonadales; Chlamydomonadaceae; Chloromonas
3	ANE33	8	AJ867742	Uncultured chlorophyte alga, clone JFJ-WS-Uni21	99	Viridiplantae; Chlorophyta; environmental samples
4	ANE58	36	AF514401	Glacier algae *Chloromonas* cf. *platystigma* 020-99	100	Viridiplantae; Chlorophyta; Chlorophyceae; Chlamydomonadales; Chlamydomonadaceae; Chloromonas
5	ANE59	8	AJ867631	Uncultured Chytridiomycete, clone WS 10-E15	99	Fungi; Chytridiomycota; environmental samples
6	ANE66	8	AJ867718	Uncultured phototrophic eukaryote, clone RS 8-Uni56-B	92	Viridiplantae
7	ANE67	4	AJ867634	Uncultured Cercozoan, clone BS 7-E06	96	Rhizaria; Cercozoa; environmental samples
8	ANE71	2	AF164272	*Rhizophlyctis harderi* isolate AFTOL-ID 31	96	Fungi; Chytridiomycota; Chytridiomycetes; Spizellomycetales; Spizellomycetaceae; Rhizophlyctis
9	ANE72	4	AY218121	*Rotaria rotatoria*	99	Metazoa; Rotifera; Bdelloidea; Rotaria
10	ANE79	8	AY965866	Soil flagellate AND21	97	Rhizaria; Cercozoa; Cercomonadida; Heteromitidae
11	MAL1	1	AF289921	Unidentified fungus sp. Kmlps3-26	96	Fungi; environmental samples
12	MAL2	5	AJ867742	Uncultured chlorophyte alga, clone JFJ-WS-Uni21	99	Viridiplantae; Chlorophyta; environmental samples
13	MAL3	12	AF514401	Glacier algae *Chloromonas* cf. *platystigma* 020-99	97	Viridiplantae; Chlorophyta; Chlorophyceae; Chlamydomonadales; Chlamydomonadaceae; Chloromonas
14	MAL4	2	AJ867634	Uncultured Cercozoan, clone BS 7-E06	96	Rhizaria; Cercozoa; environmental samples
15	MAL5	2	AY218121	*Rotaria rotatoria*	96	Metazoa; Rotifera; Bdelloidea; Rotaria
16	MAL6	11	AM114804	Uncultured Cercozoan clone WIM44	98	Rhizaria; Cercozoa
17	MAL7	2	AJ867629	Uncultured Chytridiomycete, clone WS 10-E02	98	Fungi; Chytridiomycota; environmental samples
18	PER25	20	AM114804	Uncultured Cercozoan clone WIM44	97	Rhizaria; Cercozoa
19	PER41	18	AF448477	*Raphidonema nivale*	98	Viridiplantae; Streptophyta; Klebsormidiophyceae; Klebsormidiales; Elakatotrichaceae; Raphidonema
20	PER44	16	AY496043	*Heteromita globosa* strain SCCAP H251	99	Rhizaria; Cercozoa; Cercomonadida; Heteromitidae; Heteromita
21	PER46	4	AF395497	*Chlamydomonas nivalis*	97	Viridiplantae; Chlorophyta; Chlorophyceae; Chlamydomonadales; Chlamydomonadaceae; Chlamydomonas
22	PER49	16	AJ311569	*Koliella antarctica*	100	Viridiplantae; Streptophyta; Klebsormidiophyceae; Klebsormidiales; Klebsormidiaceae; Koliella
23	PER50	20	AY965866	Soil flagellate AND21	99	Rhizaria; Cercozoa; Cercomonadida; Heteromitidae
24	PER58	12	DQ211596	*Bodomorpha* sp. HFCC57	97	Rhizaria; Cercozoa; Cercomonadida; Heteromitidae; Bodomorpha
25	PER60	2	AJ867745	Uncultured chrysophyte, clone JFJ-ICE-Uni-10	99	Stramenopiles; Chrysophyceae; environmental samples
26	PER63	4	AJ867629	Uncultured Chytridiomycete, clone WS 10-E02	98	Fungi; Chytridiomycota; environmental samples
27	LIT1	8	HQ912653	*Ardissonea formos*	97	Stramenopiles; Bacillariophyta; Fragilariophyceae; Fragilariophycidae; Fragilariales; Fragilariaceae; Ardissonea
28	LIT2	10	AF101826	*Bensingtonia yamatoana*	99	Fungi; Dikarya; Basidiomycota; Pucciniomycotina; Agaricostilbomycetes; Agaricostilbomycetes incertae sedis; mitosporic Agaricostilbomycetidae; Bensingtonia
29	LIT3	16	FN394863	Uncultured eukaryote 01DPZ110600082	98	Rhizaria; Cercozoa
30	LIT4	8	GQ503607	*Brachionus calyciflorus*	99	Metazoa; Rotifera; Monogononta; Ploimida; Brachionidae; Brachionus
31	LIT5	2	DQ104080	Uncultured Chlorophyta P200E-4	97	Viridiplantae; Chlorophyta

**Table 3:** Diversity of small eukaryotes in clone libraries from glaciers.

Taxon	Mean no. of OTUs (clones) in glacier			
	ANE	MAL	PER	LIT
Viridiplantae	4 (80)	2 (17)	3 (38)	1 (2)
Streptophyta			2 (34)	
Chlorophyta	3 (72)	2 (17)	1 (4)	1 (2)
Environmental samples	1 (8)			
Stramenopiles			1 (2)	1 (8)
Chrysophyceae			1 (2)	
Bacillariophyta				1 (8)
Fungi	3 (12)	2 (3)	1 (4)	1 (10)
Chytridiomycota	2 (10)	1 (1)	1 (4)	
Dikarya				1 (10)
Environmental samples	1 (2)	1 (1)		
Rhizaria	2 (12)	2 (13)	4 (68)	1 (16)
Cercozoa	2 (12)	2 (13)	4 (68)	1 (16)
Metazoa	1 (4)	1 (2)		1 (8)
Rotifera	1 (4)	1 (2)		1 (8)

### COMMUNITY COMPOSITION AND STATISTICAL ANALYSIS: SPECIES RICHNESS, ABUNDANCE, AND DIVERSITY

The microbial communities in higher glaciers differed from the lower glacier communities. Regardless of the chemical composition of ice (**Table 1**), this difference was mainly due to the large numbers of Viridiplantae and Rhizaria among others in higher glaciers. There was also a marked decrease in the number of clones, number of OTUs and the Shannon index of diversity index in lower glaciers (**Table 4**). Comparisons of both, the mean number of clones and the mean number of OTUs demonstrated the similarity between ANE and PER (**Table 4**). Further, the mean *H*′ which ranged from 1.565 to 2.017, revealed that the highest microbial diversity was found in PER, followed by ANE (**Table 4**).

**Table 4:** Number of clones, number of OTUs, and diversity index for the samples from Pyrenean glaciers.

Glacier	Mean no. of clones	Mean no. of OTUs	Diversity index	*H*′
ANE	112	10	1.832
MAL	35	7	1.649
PER	108	9	2.017
LIT	44	5	1.565
*r*^2^	0.9905***	0.9688**	0.9995***
Multiple	ANE vs. PER^NS^	ANE vs. PER^NS^	ANE vs. PER***
comparison	ANE vs. LIT***	ANE vs. LIT**	ANE vs. LIT***
	PER vs. LIT***	PER vs. LIT**	PER vs. LIT***
	MAL vs. PER***	MAL vs. PER*	MAL vs. PER***
	MAL vs. ANE***	MAL vs. ANE*	MAL vs. ANE***
	LIT vs. MAL^NS^	LIT vs. MAL**	LIT vs. MAL***

Statistical differences were studied by ANOVA test on the number of clones, number of OTUs and Shannon–Wiener index (**, *p* ≤ 0.001; ***, *p* ≤ 0.0001). Statistical significance was achieved by Newman-Keuls post-test (NS, not significant; *, *p* ≤ 0.05; **, *p* ≤ 0.01; ***, *p* ≤ 0.001).

Test richness, abundance, and diversity were performed by ANOVAs (**Table 4**). Subsequently, Newman–Keuls *post hoc* test was used to test for differences among samples. Statistics indicated that significant differences existed among glaciers. ANE had the highest number of clones (112) and OTUs (10) whilst PER showed 9 OTUs with 108 clones, and MAL and LIT contained 7 and 5 OTUs with 35 and 44 clones, respectively. In general, glaciers located at higher altitude and area (ANE, MAL, and PER) were more diversely populated than LIT.

**FIGURE 3:**
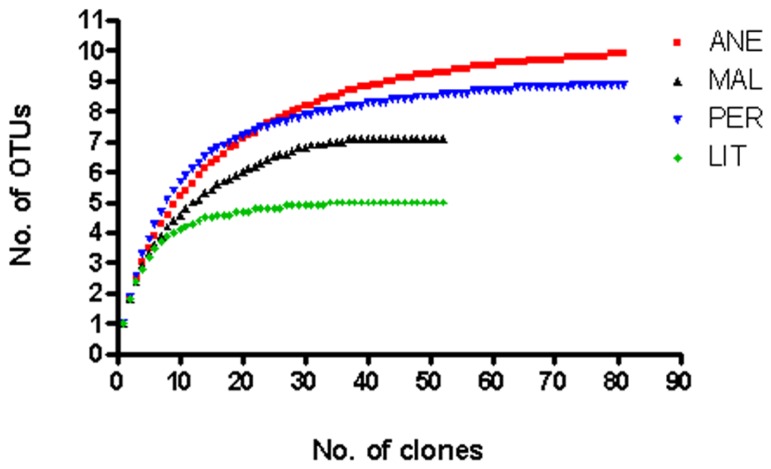
**Rarefaction curves determined for the different 18S rRNA gene clones**. Rarefaction curves indicating the observed number of operational taxonomic units (OTUs) at a genetic distance of 3% in four different glaciers.

**FIGURE 4:**
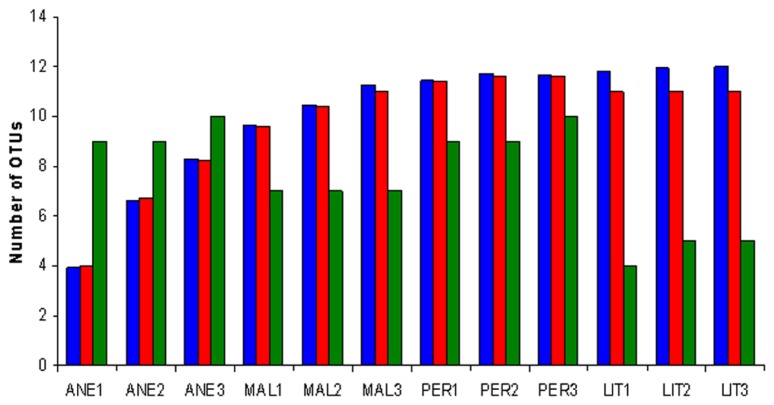
**Richness estimates of Pyrenean glaciers at a genetic distance of 3%**. Richness has been estimated by the richness estimator Chao1, which is a non-parametric richness estimator based on distribution of singletons and doubletons (blue), by the abundance-based coverage estimator (ACE), which is a non-parametric richness estimator based on distribution of abundant (>10) and rare (<10) OTUs (red), and by the number of OTUs (green). Sample numbers are given below the graph. A description of the samples is shown in **Table 2**.

Rarefaction analysis was performed. At 3% sequence divergence rarefaction curves reached saturation, indicating that the surveying effort covered almost the full extent of taxonomic diversity at this genetic distance (**Figure 3**). Comparison of the rarefaction analyses with the number of OTUs determined by Chao1 and ACE richness estimators revealed that 50–100% (3% genetic distance) of the estimated taxonomic richness was covered by the surveying effort (**Figure 4**). Thus, we did not survey the full extent of taxonomic diversity, but a substantial fraction of the microbial diversity within individual glacier samples was assessed. The comparison of mean Chao1 richness estimates in glaciers employing one-way ANOVA showed different values between ANE and the rest of glaciers (*p* = 0.0013). MAL, PER, and LIT did not vary significantly (*p* > 0.05).

**FIGURE 5:**
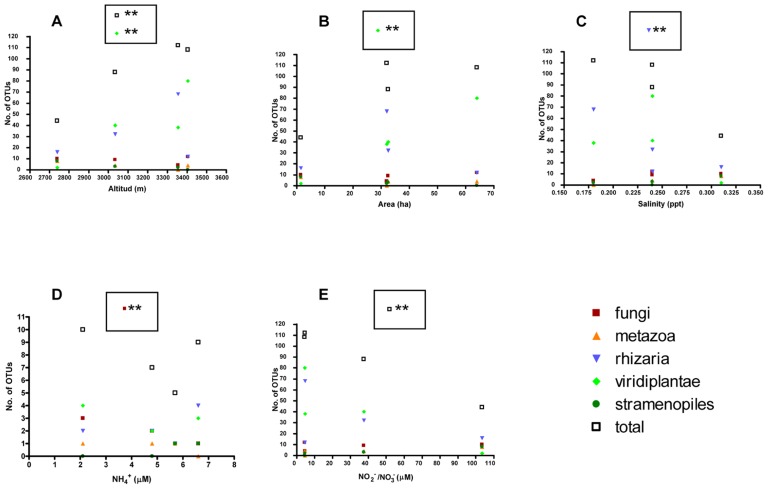
**Correlation analysis between protist taxa from glaciers and environmental variables**. Number of OTUs in glaciers with respect to environmental parameters: **(A)** Altitude; **(B)** area; **(C)** salinity; **(D)** ${NH}_{4}^{+}$ ; **(E)** $NO_{2}^{-}$/$NO_{3}^{-}$ ; statistical differences were studied by Pearson’s *r* (*p < 0.05 and **p < 0.01).

Further, several correlation analysis between protist taxa from glaciers and environmental variables demonstrated statistical differences in the total number of taxa related to the altitude (*p* = 0.0074) and also related to the number of Viridiplantae (*p* = 0.0346; **Figure 5**; **Table 5**). Other variables such as salinity and ${NH}_{4}^{+}$ showed a negative correlation in Rhizaria (*p* = 0.0203) and Fungi (*p* = 0.0165), respectively. $NO_{2}^{-}$ and $NO_{3}^{-}$ also presented a negative correlation in the total number of OTUs (*p* = 0.0424 and 0.0432), respectively.

**Table 5:** Correlation analysis between taxa of small eukaryotes from glaciers and environmental variables.

Taxon	Altitude	Area	Salinity	pH	${NH}_{4}^{+}$	$NO_{2}^{-}$	$NO_{3}^{-}$
**Fungi**
*r*	0.4981	0.8541	-0.04913	0.8843	-0.9670	-0.4966	-0.4940
*p*	0.5019	0.1459	0.4754	0.1157	0.0165*	0.5034	0.5060
**Metazoa**
*r*	-0.4777	0.0033	0.7839	0.8467	-0.6172	0.4673	0.4697
*p*	0.5223	0.9967	0.1080	0.1533	0.1914	0.5327	0.5303
**Rhizaria**
*r*	0.7167	0.3152	-0.9594	-0.6392	0.3679	-0.7460	-0.7480
*p*	0.2833	0.6848	0.0203*	0.3608	0.3161	0.2540	0.2520
**Viridiplantae**
*r*	0.9654	0.9532	-0.6558	0.2951	-0.5976	-0.9144	-0.9132
*p*	0.0346*	0.0468*	0.1721	0.7049	0.2012	0.0856	0.0868
**Stramenopiles**
*r*	-0.3209	-0.6954	0.05431	-0.8066	0.8018	0.4118	0.4099
*p*	0.6791	0.3046	0.4728	0.1934	0.0991	0.5882	0.5901
**Total**
*r*	0.9926	0.9223	-0.7566	0.1718	-0.4871	-0.9576	-0.9568
*p*	0.0074**	0.0777	0.1217	0.8282	0.2564	0.0424*	0.0432*

Statistical differences were studied by Pearson’s r (*p ≤ 0.05; **p ≤ 0.01).

### DISTRIBUTION OF TAXA AND PHYLOTYPES ACROSS ALL SAMPLES

**FIGURE 6:**
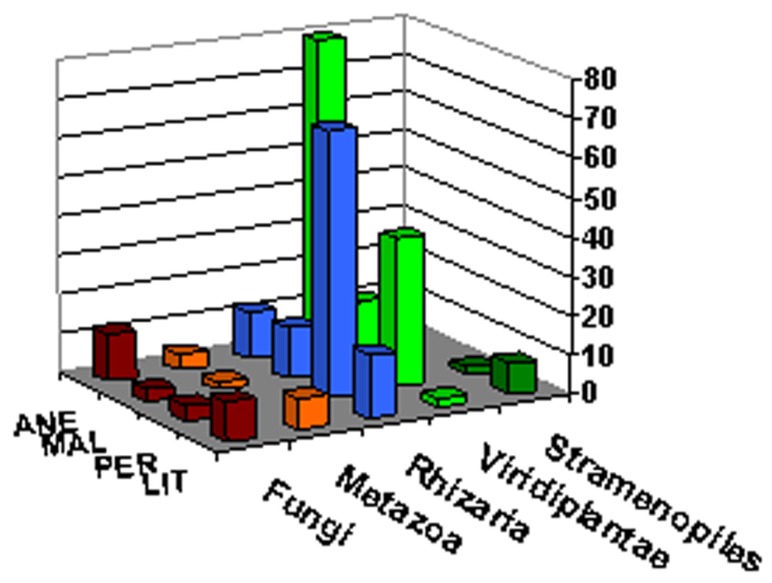
**Microbial eukaryotic diversity**. Relative 18 rRNA abundance of major eukaryotic groups in gene libraries from ice samples.

All the sequences were affiliated to five phyla, Fungi, Metazoa, Rhizaria, Viridiplantae, and Stramenopiles, representing 9.7, 4.7, 36.5, 45.8, and 3.34%, respectively (**Figure 6**). The dominant taxa were represented in all samples with the exception of Metazoa in PER and Stramenopiles in ANE and MAL. The members of rare phyla (<1% of all classified sequences) included a member of the family Chrysophyceae in PER and a member of Viridiplantae in LIT samples (**Table 2**). The most abundant phylotype across all samples was a glacier algae in ANE and MAL samples, *Chloromonas platystigma*, representing 16% of all sequences. The most abundant phylotypes in PER and LIT were two uncultured Cercozoan representing 6 and 5% of all sequences, respectively (**Table 2**).

At the genus level, comparison of the relative abundances revealed significant differences between glaciers. *Chloromonas* was the most abundant genus across all ice samples, representing 22% of all classified sequences in ANE and 4% in MAL. The distribution of the other dominant genera *Raphidonema* (4.6%), *Heteromita* (5.3%), *Koliella* (5.4%), and *Bodomorpha* (4%) varied significantly between glaciers (*p* < 0.0001; **Table 2**).

### DIFFERENCES IN COMMUNITY STRUCTURE BETWEEN GLACIERS

The relative abundances of dominant taxa varied between glaciers. Our clone libraries were clearly dominated by Viridiplantae in ANE and MAL where they represented nearly 80%. Rhizaria, especially represented by the Cercozoa in PER (**Figure 6**) and sequences affiliating to Rhizaria were also the most abundant in LIT library.

**FIGURE 7:**
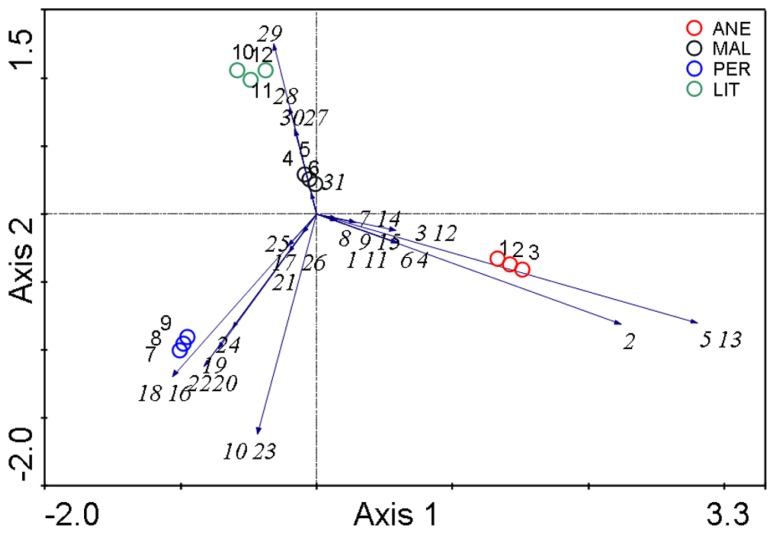
**Comparison of the microbial communities using the PCA analysis**. Numbers represent phylotypes as shown in **Table 2** and **Figure 8**. The diagram display circles representing sampling sites and arrows that indicate the direction of increase for the abundances.

Principal components analysis (PCA; analysis no. 1) based on the relative abundances of the microbial phyla confirmed that microbial communities in glaciers were quite different (**Figure 7**). MAL contained the only samples that shared similar composition with those from ANE and PER. ANE and PER contained only one common species (a soil flagellate belonging to Cercozoa), and LIT appears to be the most different.

**FIGURE 8:**
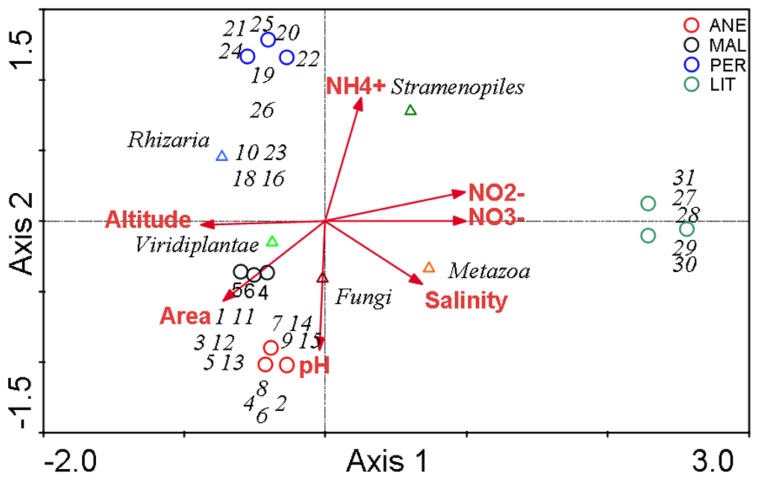
**Canonical correspondence analysis**. Ordination diagram based on CCA, with respect to seven quantitative variables. The axes are scaled in standard deviation units. Eigenvalues for the axes are detailed in **Table 6**. The diagram display triangles that represent phyla, circles representing sampling sites, and arrows that symbolize environmental variables.

Canonical correspondence analysis with all environmental variables was used to estimate the proportion of the community variability attributable to variability in the environment, which was estimated in several runs, each with a single variable. The eigenvalues corresponding to the four ordination axes were used to characterize the results of particular analysis (**Table 6**). CCA diagrams show the interrelationships between microbial communities and environmental variables that were observed in the four glaciers (**Figure 8**). The OTUs and sampling points mutually portray the dominant patterns in community composition to the extent that these could be elucidated by the selected variables (Jongman et al., 1995). The length of an arrow representing an environmental variable was considered to be equal to the rate of change in the score as inferred from **Figure 8**, hence a measure of how much the microorganism distribution differ along that variable.

**Table 6:** Summary of correspondence analysis and eigenvalues (λ).

No. of analysis	Type of analysis	Environmental variables	Environmental covariables	λ_1_	λ_2_	λ_3_	λ_4_
1	PCA	–	–	0.612	0.310	0.074	0.003
2	CCA	Altitude, area, $NO_{2}^{-}$ , $NO_{3}^{-}$, ${NH}_{4}^{+}$ , pH, salinity	–	1.000	0.798	0.200	0.003
3	CCA	Altitude, area	–	0.835	0.780	0.383	0.006
4	CCA	Altitude, area, pH	–	1.000	0.798	0.200	0.003
5	CCA	Altitude, area	pH	0.842	0.094	0.323	0.005
6	CCA	Altitude, area, $NO_{2}^{-}$	–	0.996	0.798	0.198	0.007
7	CCA	Altitude, area	$NO_{2}^{-}$	0.798	0.198	0.007	0.005

In the analysis no. 2, the CCA produced four axes which accounted for 100% of the total variance in abundances of microbial OTUs among the glaciers. **Figure 8** shows a biplot diagram of OTUs, glaciers, and environmental variables. The forward selection of variables demonstrated that the relationship between microbial communities and altitude (*p* = 0.02), area (*p* = 0.02), pH (*p* = 0.02), $NO_{2}^{-}$ (*p* = 0.046), and $NO_{3}^{-}$ (*p* = 0.048) were significant. The CANOCO program excluded ${NH}_{4}^{+}$ and salinity because they exhibited negligible variance. Subsequent analysis also demonstrated that altitude, area, and pH can explain the total community variability (analysis no. 3 and 4), meanwhile $NO_{2}^{-}$ and $NO_{3}^{-}$ are not so relevant (analysis no. 5, 6, and 7).

In summary, significant differences of the community structure between the four glaciers were visible. The comparison of relative abundances at the level of phyla also revealed significant differences (**Figure 6**; **Table 4**). In general, Viridiplantae, Fungi, and Rhizaria were positively influenced by altitude, area, and pH of glaciers, and negatively by salinity and $NO_{2}^{-}$ and $NO_{3}^{-}$ contents. These environmental characteristics were dominant in higher glaciers (**Figure 8**). On the contrary, samples collected at low altitudes, in LIT, contained a majority of Stramenopiles and Metazoa, and were positively influenced by salinity and ${NH}_{4}^{+}$ , $NO_{2}^{-}$ , and $NO_{3}^{-}$ contents (**Figure 8**).

## DISCUSSION

Among the organisms that have successfully colonized extreme cold environments, a variety of survival mechanisms have been exploited. Microbial activity in ice is restricted to small amounts of unfrozen water inside the permafrost soil or the ice, and to brine channels. While there are sparse communities of lichens, mosses, and soil microorganisms, the limited availability of liquid water curtails biological activity for most of the year (Laybourn-Parry, 2002). Glaciers have truncated food chains with no animals or plants and a dominance of protozoa, bacteria, fungi, and microalgae. These microorganisms may be trapped during ice formation and remain inactive and frozen, but also active microorganisms live within the ice, being subjected to strong physical and chemical constraints. Contrary to what one might suppose, many of the microorganisms in glaciers do not cease to function in the winter months. Bacteria continue to grow all year, showing cycles that appear to be related to the availability of dissolved organic carbon. They provide potential energy for a spectrum of heterotrophic and mixotrophic protozoans (Heath, 1988; Bell and Laybourn-Parry, 1999).

In cold environments, organisms are confronted by continuous low temperatures as well as a nutrient limitation. When compared to other known microorganisms, psychrophiles possess many unique qualities and molecular mechanisms that allow their adaptation to cold environments (Alcazar et al., 2010). In order to maintain activity in winter, microorganisms adopt one or more of a variety of strategies that enable them to enter the summer with actively growing populations. In this regard, some bacteria have been found to contain polyunsaturated fatty acids in their plasma membranes, which generally do not occur in other organisms. Further, some of them use enzymes that continue to function at near freezing ambient temperatures. And finally, they are able to produce proteins that are stable at cold temperatures (García-Descalzo et al., 2011). Among the Protozoa, many of the most successful species survive the winter in an active state by using endogenous energy reserves or employing nutritional versatility. Mixotrophy is also an important nutritional strategy (Laybourn-Parry, 2002). It involves a combination of autotrophy and heterotrophy in varying degrees. Some protozoa are forced to sustain a mixotrophic strategy and cannot survive by photosynthesis alone. The dependence on ingesting bacteria varies seasonally. One argument suggests that it is a means of acquiring inorganic nutrients for photosynthesis during phases of limitation (Nygaard and Tobiesen, 1993). Other researchers contend that it is a means of supplementing the carbon budget (Jones et al., 1993).

According to our results, the taxonomic affiliation of the eukaryotic sequences associated to the samples from higher Pyrenean glaciers was markedly different from that of lower glacier. Ice in samples from higher glaciers was characterized by a clear dominance of Viridiplantae, fundamentally *Chloromonas*, and Rhizaria (**Figures 5** and **8**) and these glaciers were quite similar in terms of relative abundance of phyla with typical glacier protist lineages, most notably Chlorophyta, Streptophyta, and Cercozoa (Cameron et al., 2012). Generally the less thawed areas, located in ANE, present a majority of Viridiplantae. Probably, ice maintains microorganisms isolated in clusters where they must survive as photosynthetic and primary producers. However, frequently thawed areas host a majority of Rhizaria that are heterotroph. In this case, microorganisms are able to move into the meltwater, reaching their preys. It is interesting to comment that although PER conserves an intermediate extension of ice, it presents a majority of Rhizaria, what could be due to the high level of fragmentation observed in the mass of ice which originates freeze-thaw cycles.

In this study, the dominant taxa were present in all samples, and corresponded roughly with those reported in other studies regarding protist community composition in ice samples (Bachy et al., 2011). In general, our knowledge about microbial eukaryotic communities in glaciers is quite limited. There are not many similar reports to compare, but overall abundance and biodiversity in our samples appear to be low, which may be due to the structure of ice, that keeps the microorganisms isolated. LIT is the only one with representatives of all phyla, since its deglaciation may allow greater exchange of microorganisms. Samples were collected in summer, when iced areas were minimal, and LIT virtually becomes a lake.

The retreat of glaciers is related to their geographical features such as altitude and area, while other environmental variables such as pH is dependent of mineral salts solubility, which in turn increases with temperature. Moreover, pH may depend on the chemical composition of the soil. We cannot determine whether pH has a direct or indirect effect on community composition, as a number of ice properties (e.g., salinity) are directly or indirectly related to pH. Thus, the effect of a number of different factors is reflected by ice pH and these factors may also drive community composition. Further, PER is placed in a calcareous massif, while the other three glaciers are located in a granite massif (Arenillas et al., 2008). However, in terms of biodiversity, the three highest glaciers are similar, regardless of their geochemical characteristics.

Climate change is one of the most important problems that concern modern society. Until now, scarce data are available to evaluate the environmental impact on living organisms due to the climate change. It is necessary to develop a rigorous investigation before undertaking any intervention, and that is why accurate data are needed about possible responses from living organisms to climate change. It is important to know how microbial patterns are being altered and how these changes are affecting to climate, as these microorganisms are essential components of the microbial food webs and are often dominant primary producers. Although the full range of ecological implications remains poorly understood, changes at the base of food webs necessarily entail consequences for higher trophic levels, while modifying the biochemical cycling of major elements including, but not limited to carbon, nitrogen, phosphorous, and silicon (Hobbs et al., 2010). The evaluation of the effect of global warming at the microbial community level is a difficult task because of the many mutually dependent response variables, non-linear responses, etc. These difficulties can be partially overcome using CCA, which enables an evaluation of the influence of the environment on the composition of the community and provides a distribution-free Monte Carlo test of significance (Jongman et al., 1995). The analysis of protist diversity in Pyrenean glaciers revealed statistically significant differences in protist diversity and community structure between four glaciers melted to different extent. The analysis of influences of ice chemical properties on protist community structure revealed that pH had the strongest effect on protist community structure of the analyzed ice properties. $NO_{2}^{-}$ and $NO_{3}^{-}$ contents appear to have a minor impact on psychrophilic protist community structure and diversity. In this survey, the correlations between ice melting and community composition were obvious.

The effect of altitude and glacier area on the composition of the microbial community is essential, as demonstrated by CCA analysis (**Figure 8**). This analysis also demonstrated that it is effectively possible to discriminate between the effect on microbial community of area/altitude and the effect of chemical parameters. Chemical composition differences appear associated to ice melting in lower glaciers, in which $NO_{2}^{-}$ and $NO_{3}^{-}$ contents are correlated to community composition.

Further work is needed to evaluate whether our observations can be generalized to other glacial regions. The retreat of mountain glaciers subsequent to ongoing climate change has been documented extensively in the last years (Meier, 1984; Gómez et al., 2003; Paul et al., 2004; Zemp et al., 2006; Citterio et al., 2007). Several studies developed analytical models to forecast the retreating trend of glaciers in the future. They predicted that a consistent loss (or even the complete extinction) of most of the ice masses will be observed by the end of this century in which case should the current climate trend continue, and concluded that small glaciers of southern Europe will be among the most reliable witnesses of global warming (Oerlemans, 1997; Zuo and Oerlemans, 1997; Zemp et al., 2006). Accordingly, such southern glaciers can be considered important for studying climate and environmental changes occurring in the Mediterranean region.

Future studies will be useful to better understand the impact of global warming on microbial communities that behave as biosensors. In our results, two main groups appear to be indicators of glacier retreat, Stramenopiles and Metazoa, meanwhile Viridiplantae is the dominant group in the less melted glaciers. There are still many unanswered questions relating to the physiology and biochemistry of psychrophilic protists that beg to be addressed. These eukaryotic microorganisms in Pyrenean glaciers raise the question of whether they have been preserved in an inactive state or were able of maintenance their metabolism for long periods of time in cold glacier environments. We also need information on the species diversity, as well as much more data on biochemistry of surviving the cold and adaptation to warming, particularly as there is a huge biotechnological potential in such psychrophiles.

Canonical correspondence analysis (CCA) was performed to analyze the relationships between the observed taxa in **Table 2** and environmental variables in **Table 1**. Seven environmental variables were taken into account. Two of them, altitude and glacier area are related to the glacier degree of conservation, as ice melting is less dramatic in higher glaciers, which maintain wider areas of ice. The rest of environmental variables, pH, salinity, ${NH}_{4}^{+}$ , $NO_{2}^{-}$ , and $NO_{3}^{-}$ are associated to the ice chemical composition. To separate the sources of variability in the microbial community various combinations with different environmental variables were applied (**Table 6**).

## Conflict of Interest Statement

The authors declare that the research was conducted in the absence of any commercial or financial relationships that could be construed as a potential conflict of interest.

## Footnotes

1 http://ncbi.nlm.nih.gov/BLAST

2 www.uga.edu/~strata/software/Software.html

3 http://purl.oclc.org/estimates

## Abbreviations

ANE: Aneto glacier
CCA: canonical correspondence analysis
LIT: Literola glacier
MAL: Maladeta glacier
PCA: principal components analysis
PCR: polymerase chain reaction
PER: Monte Perdido glacier.

## References

[B1] Alcazar A. Garcia-Descalzo L. Cid C. (2010). *Microbial Evolution and Adaptation in Icy Worlds*. New York: Nova Science Publishers/Springer Verlag Inc

[B2] Amann R. I. Binder B. J. Olson R. J. Chisholm S. W. Devereux R. Stahl D. A. (1990). Combination of 16S rRNA-targeted oligonucleotide probes with flow cytometry for analyzing mixed microbial populations. *Appl. Environ. Microbiol.* 56 1919–1925 2200342 10.1128/aem.56.6.1919-1925.1990 PMC184531

[B3] Arenillas M. Cobos G. Navarro J. (2008). *Datos sobre la nieve y los glaciares en las cordilleras españolas. El programa ERHIN (1984–2008)*. Madrid: Ministerio de Medio Ambiente y Medio Rural y Marino

[B4] Bachy C. López-García P. Vereshchaka A. Moreira D. (2011). Diversity and vertical distribution of microbial eukaryotes in the snow, sea ice and seawater near the north pole at the end of the polar night. *Front. Microbiol.* 2 106 10.3389/fmicb.2011.00106 PMC3153057 21833337

[B5] Bell E. M. Laybourn-Parry J. (1999). Annual plankton dynamics in an Antarctic saline lake. *Freshw. Biol.* 41 507–519

[B6] Beniston M. Diaz H. F. Bradley R. S. (1997). Climatic change at high elevation sites: an overview. *Clim. Change* 36 233–251

[B7] Bidle K. D. Lee S. Marchant D. R. Falkowski P. G. (2007). Fossil genes and microbes in the oldest ice on Earth. *Proc. Natl. Acad. Sci. U.S.A.* 104 13455–13460 17686983 10.1073/pnas.0702196104 PMC1941643

[B8] Branda E. Turchetti B. Diolaiuti G. Pecci M. Smiraglia C. Buzzini P. (2010). Yeast and yeast-like diversity in the southernmost glacier of Europe (Calderone Glacier, Apennines, Italy). *FEMS Microbiol. Ecol.* 72 354–369 20402775 10.1111/j.1574-6941.2010.00864.x

[B9] Butinar L. Spencer-Martins I. Gunde-Cimerman N. (2007). Yeasts in high Arctic glaciers: the discovery of a new habitat for eukaryotic microorganisms. *Antonie Van Leeuwenhoek* 91 277–289 17072534 10.1007/s10482-006-9117-3

[B10] Cameron K. A. Hodson A. J. Osborn A. M. (2012). Structure and diversity of bacterial, eukaryotic and archaeal communities in glacial cryoconite holes from the Arctic and the Antarctic. *FEMS Microbiol. Ecol.* 82 254–267 22168226 10.1111/j.1574-6941.2011.01277.x

[B11] Caron D. A. Peele E. R. Lim E. L. Dennett M. R. (1999). Picoplankton and nanoplankton and their trophic coupling in the surface waters of the Sargasso Sea south of Bermuda. *Limnol. Oceanogr.* 44 259–272

[B12] Chao A. Bunge J. (2002). Estimating the number of species in a stochastic abundance model. *Biometrics* 58 531–539 12229987 10.1111/j.0006-341x.2002.00531.x

[B13] Christner B. C. Mikucki J. A. Foreman C. M. Denson J. Priscu J. C. (2005). Glacial ice cores: a model system for developing extraterrestrial decontamination protocols. *Icarus* 174 572–584

[B14] Citterio M. Diolaiuti G. Smiraglia C. D’Agata C. Carnielli T. Stella G. (2007). The fluctuations of Italian glaciers during the last century: a contribution to knowledge about Alpine glacier changes. *Geogr. Ann. A.* 89 164–182

[B15] Colwell R. K. (2011). EstimateS: Statistical estimation of species richness and shared species from samples. (Software and User’s Guide), Version 8.2.0. Available at: http://viceroy.eeb.uconn.edu/estimates

[B16] de García V. Brizzio S. Libkind D. Buzzini P van Broock M. (2007). Biodiversity of cold-adapted yeasts from glacial meltwater rivers in Patagonia (Argentina). *FEMS Microbiol. Ecol.* 59 331–341 17313582 10.1111/j.1574-6941.2006.00239.x

[B17] Djukic I. Zehetner F. Watzinger A. Horacek M. Gerzabek M. H. (2012). In situ carbon turnover dynamics and the role of soil microorganisms therein: a climate warming study in an Alpine ecosystem. *FEMS Microbiol. Ecol.* 83 112–124 22809312 10.1111/j.1574-6941.2012.01449.x

[B18] Dolhi J. M. Ketchum N. Morgan-Kiss R. M. (2012). Establishment of microbial eukaryotic enrichment cultures from a chemically stratified Antarctic lake and assessment of carbon fixation potential. *J. Vis. Exp. * 62 pii:3992 10.3791/3992 PMC3466643 22546995

[B19] Foght J. M. Aislabie J. Turner S. Brown C. E. Ryburn J. Saul D. J. (2004). Culturable bacteria in subglacial sediments and ice from two southern hemisphere glaciers. *Microb. Ecol.* 47 329–340 14994176 10.1007/s00248-003-1036-5

[B20] García-Descalzo L. Alcazar A. Baquero F. Cid C. (2011). Identification of in vivo HSP90-interacting proteins reveals modularity of HSP90 complexes is dependent on the environment in psychrophilic bacteria. *Cell Stress Chaperones* 16 203–218 20890740 10.1007/s12192-010-0233-7 PMC3059794

[B21] Gilichinsky D. Wagene S. (2006). Microbial life in permafrost: a historical review. *Permafrost Periglac. Process.* 6 243–250

[B22] Gómez A. Palacios D. Luengo E. Tanarro L. M. Schulte L. Ramos M. (2003). Talus instability in a recent deglaciation area and its relationship to buried ice and snow cover evolution (Picacho del Veleta, Sierra Nevada, Spain). *Geogr. Ann. A* 85 165–182

[B23] Heath C. W. (1988). Annual primary production of an Antarctic continental lake: phytoplankton and benthic algal mat production strategies. *Hydrobiologia* 165 77–87

[B24] Hobbs W. O. Telford R. J. Birks H. J. B. Saros J. E. Hazewinkel R. R. O. Perren B. B. (2010). Quantifying recent ecological changes in remote lakes of North America and Greenland using sediment diatom assemblages. *PLoS ONE *5:e10026. 10.1371/journal.pone.0010026. PMC2848865 20368811

[B25] IPCC. (2007). “Climate change 2007: the physical science basis,” in *Contribution of Working Group I to the Fourth Assessment Report of the Intergovernmental Panel on Climate Change*, eds Solomon S. Qin D. Manning M. Chen Z. Marquis M. Averyt K. B. (Cambridge: Cambridge University Press) 2–19

[B26] Jones H. L. J. Leadbeater B. S. C. Green J. C. (1993). Mixotrophy in marine species of Chrysochromulina (Prymnesiophyceae): ingestion and digestion of a small green flagellate. *J. Mar. Biol. Assoc. U.K.* 73 283–296

[B27] Jongman R. H. G. Ter Braak C. J. F Van Tongeren O. F. R. (1995). *Data Analysis in Community and Landscape Ecology*. Cambridge: Cambridge University Press

[B28] Larkin M. A. Blackshields G. Brown N. P. Chenna R. McGettigan P. A. McWilliam H. (2007). Clustal W and Clustal X version 2.0. *Bioinformatics* 23 2947–2948 17846036 10.1093/bioinformatics/btm404

[B29] Laybourn-Parry J. (2002). Survival mechanisms in Antarctic lakes. *Philos. Trans. R. Soc. Lond. B Biol. Sci.* 357 863–869 12171649 10.1098/rstb.2002.1075 PMC1692993

[B30] Lefranc M. Thenot A. Lepere C. Debroas D. (2005). Genetic diversity of small eukaryotes in lakes differing by their trophic status. *Appl. Environ. Microbiol.* 71 5935–5942 16204507 10.1128/AEM.71.10.5935-5942.2005 PMC1266003

[B31] Li W. K. W. Subba Rao D. V. Harrison W. G. Smith J. C. Cullen J. J. Irwin B. (1994). Autotrophic picoplankton in the tropical ocean. *Science* 219 292–295 17798278 10.1126/science.219.4582.292

[B32] Ludwig W. Strunk O. Westram R. Richter L. Meier H. Yadhukumar, et al. (2004). ARB: a software environment for sequence data. *Nucleic Acids Res.* 32 1363–1371 14985472 10.1093/nar/gkh293 PMC390282

[B33] Ma L. Rogers S. O. Catranis C. M. Starmer W. T. (2000). Detection and characterization of ancient fungi entrapped in glacial ice. *Mycologia* 92 286–295

[B34] Margesin R. Fonteyne P. A. Schinner F. Sampaio J. P. (2007). *Rhodotorula psychrophila* sp. nov., *Rhodotorula psychrophenolica* sp. nov. and *Rhodotorula glacialis* sp. nov., novel psychrophilic basidiomycetous yeast species isolated from Alpine environments. *Int. J. Syst. Evol. Microbiol.* 57 2179–2184 17766895 10.1099/ijs.0.65111-0

[B35] Medinger R. Nolte V. Pandey R. V. Jost S. Ottenwälder B. Schlötterer C. (2010). Diversity in a hidden world: potential and limitation of next-generation sequencing for surveys of molecular diversity of eukaryotic microorganisms. *Mol. Ecol.* 19 32–40 20331768 10.1111/j.1365-294X.2009.04478.x PMC2953707

[B36] Meier M. F. (1984). Contribution of small glaciers to global sea level. *Science* 226 1418–1421 17788995 10.1126/science.226.4681.1418

[B37] Michelsen A. Rinnan R. Jonasson S. (2012). Two decades of experimental manipulations of heaths and forest understory in the subarctic. *Ambio* 41(Suppl. 3) 218–230 22864696 10.1007/s13280-012-0303-4 PMC3535062

[B38] Nygaard K. Tobiesen A. (1993). Bacterivory in algae: a survival strategy during nutrient limitation. *Limnol. Oceanogr.* 38 273–279

[B39] Oerlemans J. (1997). A flowline model for Nigardsbreen, Norway: projection of future glacier length based on dynamic calibration with the historic record. *Ann. Glaciol.* 24 382–389

[B40] Paul F. Kääb A. Maisch M. Kellenberger T. Haeberli W. (2004). Rapid disintegration of Alpine glaciers observed with satellite data. *Geophys. Res. Lett.* 31 L21402

[B41] Raup D. M. (1975). Taxonomic diversity estimation using rarefaction. *Paleobiology* 1 333–342

[B42] Rene P. (2007). Les glaciers du Parc National des Pyrénées: présentation et observations. *J. Parc Natl. Pyren.* 21 3–5

[B43] Rogers S. O. Theraisnathan V. Ma L. J. Zhao Y. Zhang G. Shin S.-G. (2004). Comparisons of protocols for examinations samples for biological and molecular decontamination of environmental ice. *Appl. Environ. Microbiol.* 70 2540–2544 15066857 10.1128/AEM.70.4.2540-2544.2004 PMC383132

[B44] Skidmore M. Foght J. M. Sharp M. J. (2000). Microbial life beneath a high arctic glacier. *Appl. Environ. Microbiol.* 66 3214–3220 10919772 10.1128/aem.66.8.3214-3220.2000 PMC92136

[B45] Tamura K. Peterson D. Peterson N. Stecher G. Nei M. Kumar S. (2011). MEGA5: molecular evolutionary genetics analysis using maximum likelihood, evolutionary distance, and maximum parsimony methods. *Mol. Biol. Evol.* 28 273–279 21546353 10.1093/molbev/msr121 PMC3203626

[B46] Tipper J. C. (1979). Rarefaction and rarefiction – the use and abuse of a method in paleontology. *Paleobiology* 5 423–434

[B47] Turchetti B. Buzzini P. Goretti M. Branda E. Diolaiuti G. D’Agata C. (2008). Psychrophilic yeasts in glacial environments of Alpine glaciers. *FEMS Microbiol. Ecol.* 63 73–83 18067577 10.1111/j.1574-6941.2007.00409.x

[B48] Wagner A. O. Hofmann K. Prem E. Illmer P. (2012). Methanogenic activities in alpine soils. *Folia Microbiol.* 57 371–373 22528315 10.1007/s12223-012-0145-2

[B49] Zemp M. Haeberli W. Hoelzle M. Paul F. (2006). Alpine glaciers to disappear within decades? *Geophys. Res. Lett.* 33 L13504

[B50] Zuo Z. Oerlemans J. (1997). Numerical modelling of the historic front variation and the future behaviour of the Pasterze glacier, Austria. *Ann. Glaciol*. 24 234–241

